# Single-cell RNA-Seq analysis identified kidney progenitor cells from human urine

**DOI:** 10.1007/s13238-020-00816-5

**Published:** 2021-01-09

**Authors:** Yujia Wang, Yu Zhao, Zixian Zhao, Dandan Li, Hao Nie, Yufen Sun, Xiaobei Feng, Ting Zhang, Yu Ma, Jing Nie, Guangyan Cai, Xiangmei Chen, Wei Zuo

**Affiliations:** 1 East Hospital, School of Medicine, Tongji University, Shanghai, 200092 China; 2 Regend Therapeutics Co. Ltd., Zhangjiang Hi-tech Park, Shanghai, 201203 China; 3 Ningxia Medical University, Yinchuan, 750004 China; 4 The First Affiliated Hospital, Guangzhou Medical University, Guangzhou, 510120 China; 5 Ruijin Hospital, School of Medicine, Shanghai Jiaotong University, Shanghai, 200025 China; 6 State Key Laboratory of Organ Failure Research, National Clinical Research Center of Kidney Disease, Key Laboratory of Organ Failure Research, Division of Nephrology, Nanfang Hospital, Southern Medical University, Guangzhou, 510515 China; 7 National Clinical Research Center for Kidney Diseases, State Key Laboratory of Kidney Diseases, Chinese PLA General Hospital, Beijing, 100039 China

**Dear Editor,**

Urine passes through the entire kidney and urinary tract system starting from the glomerulus and ending to the urethra. Cells in the kidney and urinary tract could be exfoliated from the epithelium into the urine, while leukocyte could infiltrate from the local tissue into the urine, which makes the urine a useful subject for clinical evaluation of relevant diseases. Among them, renal tubular cells and podocytes have been identified and 2D or 3D cultured from human urine specimens (Oliveira Arcolino et al., 2015; Schutgens et al., 2019). Particularly, kidney stem cell/progenitor cells were successfully recovered from pediatric patient urine and then cultured for kidney regenerative purpose by the Romagnani group. However, they also showed that such cells cannot be recovered from healthy individuals (Lazzeri et al., 2015). It remains unknown whether similar types of progenitor cells can be found in different individuals, either healthy or diseased.

The exact composition of various cell populations in human urine is poorly understood so far. The traditional histological analysis method is low-throughput and can miss some low-abundance but important cell populations. Flow cytometry used to sort cell populations from the urine provides an alternative, but it requires a priori assumptions about known cell surface markers. Recently, the fast-evolving single-cell or single-nucleus transcriptional profiling technology allows researchers to define cell types on the basis of their global transcriptomic patterns, which could help to dissect the cellular composition of the kidney (Park et al., 2018; Wu et al., 2018) as well as many other organs or biofluids.

To determine the feasibility of single-cell transcriptomic profiling of urinary cells, we performed scRNA-seq on voided urine samples. 50–100 mL middle stream urine samples were collected from 12 Chinese healthy adults and combined for droplet-based single-cell RNA sequencing after flow cytometric sorting of live cells. In total, we isolated and sequenced 2,200 cells from the urine-derived cell suspensions, and using stringent quality controls, we eventually analyzed 1,010 cells. We performed unsupervised graph-based clustering (Seurat method) of the dataset and identified 7 transcriptionally distinct cell clusters (Fig. 1A), which were visualized using t-distributed stochastic neighbor embedding (tSNE). We also tested a different clustering method (uniform manifold approximation and projection, UMAP) and identified similar cell groups (Fig. S1A).

**Figure 1:**
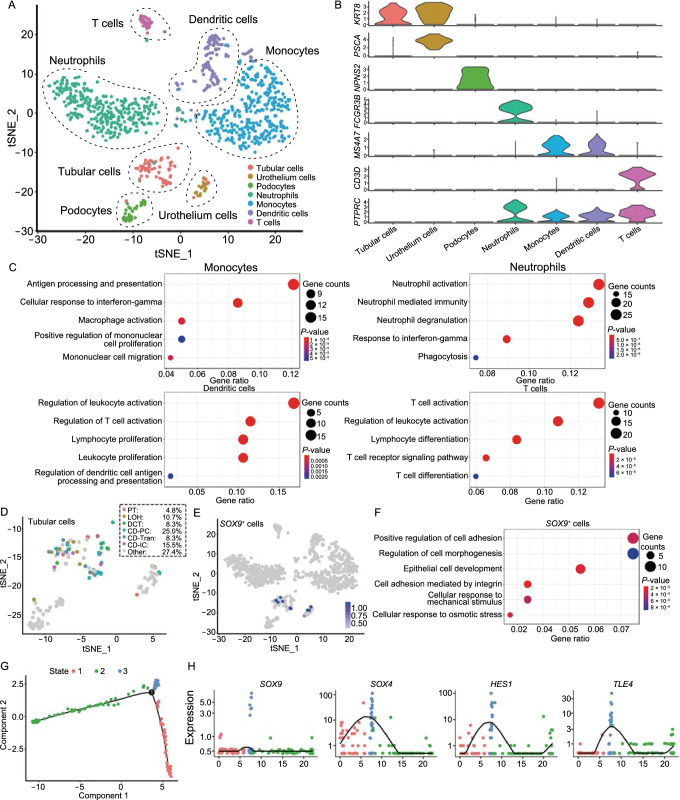
Cell identification in human urine delineated by single-cell transcriptomic analysis. (A) Unsupervised clustering demonstrating 7 distinct cell types using a t-distributed stochastic neighbor embedding (tSNE) plot. (B) Violin plots of the expression levels of representative marker genes across the 7 clusters. Among them, 4 clusters were annotated as leukocytes for their *PTPRC* (*CD45*) expression. The y axis shows the log-scale normalized read count. Tubu, tubular cells; Uroth, urothelium cells; Podo, podocytes; Neutro, neutrophils; Mono, monocytes; DC, dendritic cells; T, T cells. (C) Gene Ontology enrichment analysis of the differentially expressed genes identified in 4 leukocytes populations. (D) tSNE plot showing sub-types classified in urine-derived tubular cells. Percentages of assigned cell types in the whole tubular population are summarized in the dotted box. PT, proximal tubule; LOH, loop of Henle; DCT, distal convoluted tubule; CD-PC, collecting duct principal cell; CD-IC, collecting duct intercalated cell; CD-Trans, collecting duct transitional cell. (E) tSNE plot highlighting *SOX9* expressing cells in urine-derived cells. (F) Gene Ontology enrichment analysis of the differentially expressed genes identified in *SOX9*-expressing cells. (G) Ordering single cells along a pseudotime trajectory using Monocle2. “Tubular Cells” and “Podocytes” clusters were used for ordering and plotted in low-dimensional space. The pseudotime trajectory was divided into three different states marked by different colors. (H) Expression dynamics of *SOX9*, *SOX4*, *HES1*, and *TLE4* over pseudotime

We annotated the identity of the 7 urinary cell clusters based on the established cell type-specific markers. Violin plots and heatmap for representative differentially expressed genes from each of the populations are shown (Figs. 1B, S1B and S1C). Among them, 4 clusters were annotated as different types of leukocytes because of their *PTPRC* (*CD45*) expression. The emergence of leukocytes in urine can be an indicator of kidney and urinary tract pathological processes, and some leukocytes are also known to exist in the urine of healthy adults. However, their exact number and types in healthy adult population are previously unknown. Here we found that the most abundant leukocyte in urine is monocyte/macrophage marked by *CD14*/*CD68* (Fig. S2A). The monocyte/macrophage cell cluster accounted for 34.95% of total urinary cells. Gene Ontology enrichment analysis of the differentially expressed genes identified monocyte-specific processes relevant to antigen processing and presentation, and macrophage activation (Fig. 1C). Further investigation identified two sub-clusters of monocytes representing a non-classical *CD16*+ group and a classic *CD16*− group (Fig. S2B and S2C), which was consistent with previous single-cell studies in adult human kidney (Wu et al., 2018).

Previous studies failed to identify dendritic cells from healthy subjects (Rossi et al. 2013). However, in our urinary cell dataset, we identified a significant number of myeloid dendritic cells marked by *CD1C*/*FCER1A* (Fig. S2A). The dendritic cell cluster accounted for 12.77% of total urinary cells. Gene ontology analysis showed that cells in this dendritic cell cluster highly expressed genes related to the regulation of T cell activation and regulation of dendritic cell antigen processing and presentation (Fig. 1C). We also analyzed three MHC-II receptors (*HLA-DRB1*, *HLA-DQA1*, and *HLA-DPA1*) in urinary cells and found that their gene expression level was gradually increased from monocyte cluster to dendritic cell cluster (Fig. S2D). Therefore, it seems that the mature dendritic cells indeed exist in healthy human urine.

Functional neutrophil has been reported to exist in healthy human urine. Here in our dataset, we found that neutrophil (marked by *FCGR3B*) is another abundant cell population in the urine. This neutrophil cell cluster accounted for 32.57% of total cells. Gene Ontology enrichment analysis of the differentially expressed genes identified cell type-specific processes relevant to neutrophil activation, neutrophil degranulation and phagocytosis (Fig. 1C). No expression of other granulocyte markers was detected in this cluster.

T cell cluster (marked by *CD3D*) accounted for 3.76% of total urinary cells. Gene Ontology enrichment analysis of the differentially expressed genes identified cell type-specific processes relevant to T cell activation, T cell receptor signaling pathway and T cell differentiation (Fig. 1C). We further dissected the distinct urine-derived T cell subsets. Among all cells in this cluster, 15.8% are annotated as *CD3*+/*CD4*−/*CD8*+ cytotoxic T cells, while 55.3% are *CD3*+/*CD4*+/*CD8*− helper T cells. As the normal *CD4*/*CD8* T cell ratio in the blood is 0.9–6.0, it seems that the T cell sub-type population in urine is similar to blood.

Besides the 4 leukocyte clusters, we also identified 3 clusters of epithelial cells including podocytes (*NPHS2*+), renal tubular cells (*KRT8*+/*PSCA*−) and urothelial cells (*KRT8*+/*PSCA*+). For urine-derived podocyte, Gene Ontology enrichment analysis of the differentially expressed genes identified cell type-specific processes relevant to glomerulus development and renal filtration cell differentiation (Fig. S3A). For urine-derived tubular cells, to further dissect their exact segment localization, we compared our dataset with the previously published kidney single-cell atlas including distinct types of tubular cells from different segments. Based on the “segment gene signature” generated by that kidney single-cell dataset, we classified our urine-derived tubular cells to sub-types. The result showed that the majority (48.8%) of urine-derived tubular cells were collecting duct cells, including principal cells (PCs), intercalated cells (ICs), and the recently identified transitional cell types between PCs and ICs (Park et al., 2018). Cells from proximal tubule, loop of Henle, and distal tubule are also discovered in the urine (Fig. 1D). Similarly, we dissected that urothelial cells and found that the majority of them (80.7%) were *KRT18*+ umbrella cells, which were superficial cells covering the urothelium (Fig. S3B).

Previous studies reported the culture of different types of urinary cells for regenerative medicine purpose (Lazzeri et al., 2015; Schutgens et al., 2019). However, it has been recognized that in healthy subjects, many of the shed cells in voided urine are senescent. To understand the urinary cell viability, we performed cell cycle analysis of the single cells. The result showed that 45.3% of the analyzed cells are in the G_2_/M or S phase of the cell cycle, suggesting that about half of the living urinary cells are in non-senescent status. The leukocyte cell clusters have more non-senescent cells than the epithelial clusters. T cell cluster is the most viable cell population, and the urothelial cell cluster has most senescent cells (Fig. S3C). Altogether these data suggested that many immune cells and some epithelial cells might be proliferative and functional in the urine of healthy adults, providing the opportunity to isolate and culture such cells.

Kidney diseases pose a large threat to human health and multiple kidney pathogenesis involve progressive and inexorable destruction of renal tissue, which usually lead to renal dysfunction and might eventually result in end-stage renal diseases. Therefore, cell replacement therapy is becoming the most urgent need for kidney diseases. We have particular interest to know whether there are viable stem/progenitor cells with kidney regenerative capacity in adult human urine. Sex-determining region Y box (SOX) is a family of transcription factors that are widely involved in maintaining cells in a stem/progenitor cell-like state and inhibiting cell differentiation. Among them, SOX9 was considered as a marker of stem or progenitor population in multiple tissues, including hair bulge, airway epithelium, intestine, pancreas, and neural crest. During fetal mouse kidney development, SOX9 is expressed within the ureteric tip since the earliest stages, and is required for ureter branching as well as maintaining ureteric tip identity . In the adult mouse kidney, we and others reported that rare individual, or small clusters of SOX9+ cells exist in renal tubules. Mature tubule cells could be de-differentiated and/or activated to turn into such SOX9+ cells with progenitor-like characteristics, which could differentiate into renal tubular epithelial cells in response to acute kidney injury (Kumar et al., 2015; Kang et al., 2016). Therefore, it is becoming clear that SOX9 is a potential marker for kidney progenitor cell (KPC), at least for the mouse.

Here we characterized *SOX9*+ cells in our human urinary cell dataset. We found that 1.9% of urinary cells were *SOX9*-expressing cells. The expression of *SOX9* genes was restricted to epithelial cells—most of them distributed in the tubular cell clusters while the other few distributed in the urothelial cell clusters (Figs. 1E and S3D). Gene function enrichment analysis showed that such *SOX9*+ cells highly expressed genes that were related to epithelial cell development and regulation of cell morphogenesis, suggesting that they might have progenitor-like properties (Fig. 1F). Additionally, cell cycle analysis of the *SOX9*+ cells population indicated 31.58% of them are in the G_2_/M or S phase, and the low expressions of *P53*, *P16*, and *P21* suggested their non-senescent status in most circumstances (Fig. S3E and S3F).

We also used pseudo-time analysis to dissect the developmental relationship of tubular cells, podocytes, and putative progenitor cells. Based on unsupervised modeling, we constructed a developmental tree map, which demonstrated two trajectories (labeled as red and green color) starting from the root (labeled as blue color) (Fig. 1G). We found that podocyte marker genes were exclusively expressed in green color trajectory while the tubular marker genes were exclusively expressed in the red color trajectory, representing two directions of cell fate determination (Fig. S3G).

Interestingly, we found that *SOX9*+ cells were exclusively positioned in the blue color root, suggesting that such cells might be the ancestor of other cells. In addition to *SOX9*, we also found high expression of *SOX4*, a gene required for kidney development, in the blue color root (Huang et al., 2013). In addition, two major Notch pathway components (*HES1* and *TLE4*) also demonstrated similar expression patterns as *SOX9* and *SOX4* (Fig. 1H). Therefore, it seems that this population of *SOX9*+/*SOX4*+/Notch^high^ is primitive and could be a population of KPC in the urine.

Then we performed experiments to examine whether the SOX9+ urinary KPCs (uKPC) could give rise to tubular cells or other types of epithelial cells. First of all, we tried to isolate the SOX9+ cells from human urine. Here we introduced our previously developed 3T3 feeder cell-based “F-Clone” system plus SCM-6F8 media to selectively isolate and culture clonogenic cells from urine. In F-Clone system, the combination of growth factors and regulators of TGF-β, EGF, IGF, Wnt/β-catenin and Notch pathways support the maintenance of lung stem/progenitor cells as previously reported (Zuo et al., 2015; Ma et al., 2018). To favor renal cell growth, we have optimized the culture medium by testing different growth factor combinations. As a result, TGFβ inhibitor present in the original SCM-6F8 formulation was replaced with recombinant BMP7. The fresh urine sample was collected for each individual and urinary cells were grown in this culture system. Within 3 to 7 days, a few cells grew up rapidly and formed compact epithelial colonies. Immunostaining confirmed the expression of SOX9 in such cells, and also the expression of proliferative cell marker KI67. Human-specific nuclei antigen was also detected to confirm the human-origin of the cell colonies (Fig. 2A). To show the process that the SOX9+ uKPCs was selectively grown, the cell clones were examined from Passage 0 to Passage 8 and immunostaining on cell culture were conducted to quantify the SOX9+ cell ratio. For each urine sample, there were less than 10 cell clones were successfully isolated at Passage 0 and averagely 96% of the cells were SOX9 expressing. The SOX9+ uKPCs were then expanded while maintained their purity in the following passages (Fig. S4A and S4B). In total, we tested 9 healthy adult urine samples and successfully obtained clonogenic cells from 8 (88.8%) of them. We also tested 8 CKD patient urine samples, obtained clonogenic cells from 6 (75%) of them, and 3 of them were genetically modified by GFP-expressing lentivirus (Fig. S4C).

**Figure 2:**
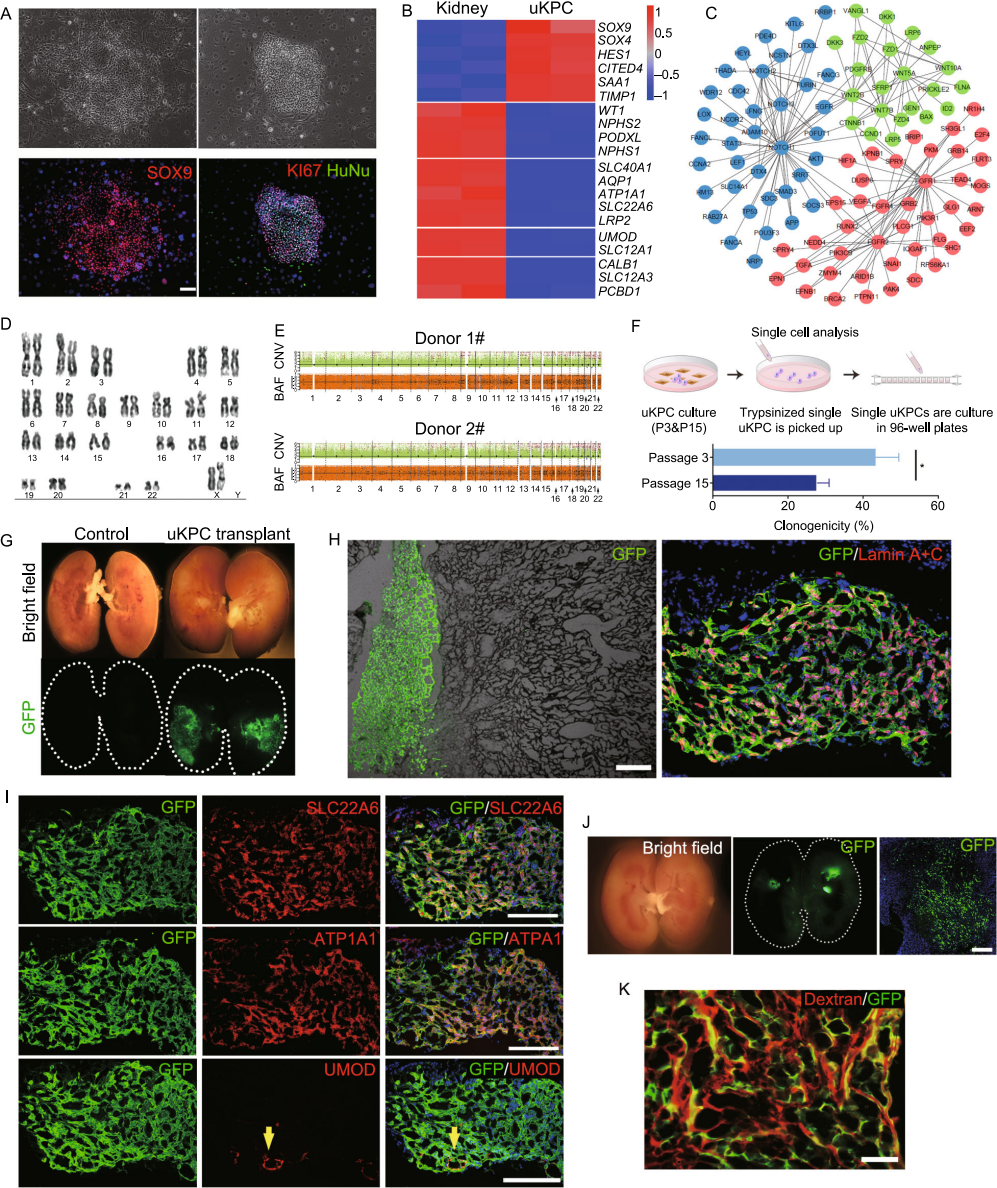
Characterization and intrarenal transplantation of human single cell-derived uKPCs. (A) Cultured human uKPC colonies stained with SOX9, KI67 and human-specific marker human nuclear antigen HuNu (representative image of *n* = 3 independent human cell pools). Scale bar, 100 μm. (B) Heatmap of differentially expressed gene sets of human kidney tissue and the cultured uKPC by RNA-seq analysis. Duplicates were taken from independent biological samples. (C) Protein-protein interaction network of selected genes with high expression levels in uKPC versus human kidney tissue. (D) Representative karyotype image of human uKPCs after 25 passages. (E) CNV and BAF profiles of 2 human uKPC lines at passage 14. Regions of copy number gain/loss and loss of heterozygosity regions were shown. Predicted BAF and copy number profiles are shown in black. Gains, losses and loss of heterozygosity are shown in red, blue and light blue dots, respectively. (F) Scheme demonstrating the single-cell picking by 96-well plates followed by quantification of single-cell clonogenicity at the early (Passage 3) and the late (Passage 15) passage of human uKPC. *n* = 3 individual clonogenic assays using independent biological samples. (G) Bright-field and direct fluorescence of injured NOD-SCID mouse kidney 14 days after saline or single cell-derived GFP+ uKPC intrarenal transplantation. Kidneys were longitudinally cut open in the midline for direct observation of the GFP signal. *n* = 10 independent transplantation experiments. uKPCs at passage 9 were used for transplantation in the representative image. (H) Representative bright field and immunostaining images on frozen sections of the transplanted kidney 14 days after transplantation. The descendants of GFP+ single cell-derived uKPC demonstrated lumen structures. uKPCs at passage 14 were used for transplantation in the representative image. Scale bar, 50 μm. Lamin A + C, a human specific nuclei antigen. (I) Frozen sections of transplanted kidney 14 days after single cell-derived uKPC transplantation followed by immunostaining with GFP and indicated tubular cell markers. Yellow arrow indicates UMOD+ GFP+ cells by immunostaining. uKPCs at passage 14 were used for transplantation in the representative image. Scale bars, 100 μm. (J) Bright-field and fluorescence of injured NOD-SCID mouse kidney 14 days after GFP+ uKPC intrarenal transplantation. The transplanted uKPCs were derived from CKD patients. *n* = 3 independent donors. Scale bar, 100 μm. uKPCs at passage 11 were used for transplantation in the representative image. (K) Rhodamine-Dextran uptake assay on the regenerated GFP+ human tubules. Scale bar, 50 μm

Bulk RNA-seq analysis of the cultured uKPCs showed that comparing to human kidney tissue, they highly express the previously identified markers in scRNA-seq including *SOX9*, *SOX4* and, *HES1*; in contrast, they did not express the mature tubular or glomerular markers (Fig. 2B). This result is consistent with the scRNA-seq analysis on primary uKPC directly isolated from urine (Fig. S3H). Our RNA-seq data allowed us to localize the expression of genes associated with specific pathways and illustrate their interaction relationship by Protein-protein interaction (PPI) network analysis. The results indicated that the uKPC highly expressed many signaling pathway component genes, which formed an interaction network centered on Notch ligand (*NOTCH1*/*NOTCH3*) and FGF receptors (*FGFR1*/*FGFR2*) and Wnt pathway components. Notch, FGF and Wnt pathways all have been previously recognized as important for kidney development and progenitor cell activation (Fig. 2C) .

The human uKPC cultures can be stably maintained *in vitro* for long-term expansion. One line in our lab has been passaged for six months and 25 generations, which could yield approximately 1 × 10^20^ cells with normal karyotype maintained (Fig. 2D). Also, whole-genome copy number variation (CNV) profiling revealed a very limited spectrum of somatic chromosomal copy number changes existed in the long-term cultured uKPC (Fig. 2E). In order to characterize the long-term propagation ability at single-cell resolution, we manually picked 100 single cells from the early (Passage 3) and the late (Passage 15) passages to compare their clonogenic capacity. Single cells cloned from early passage are morphologically identical to the late passage ones. However, during subsequent clonal plating, the average clonogenicity of cells decreased from 42% (early passages) to 23% (late passages) (Fig. 2F). This result confirmed the transit-amplifying progenitor-like properties of the cells that could replicate for long-term without spontaneous differentiation yet with gradual loss of clonogenicity (Diep et al., 2011).

In order to test whether the uKPC could give rise to tubular cells *in vivo*, we performed xeno-transplantation by injecting a single uKPC cell pedigree into the kidney of immunodeficient mice. To this end, a single uKPC cell derived from healthy donor was manually picked into 96-well plate and propagated into a single cell-derived uKPC line, which was then genetically labeled with GFP by lentiviral infection. At first, the GFP-labeled uKPCs were directly injected into the healthy mouse kidney, and no cell engraftment was observed at all a few days later. We also tried to transplant the uKPC under the renal capsule. The transplantation process was successful, however, no cell differentiation towards mature tubules was observed. These data implied that a severe local tissue injury might be a prerequisite for exogenous cell survival and differentiation (Fig. S5A and S5B). Therefore, the kidney subjected to transplantation was preconditioned by wedge resection surgery. After such surgery, all mice survived well but histological analysis demonstrated massive renal tissue injury, large-scale immune cell infiltration and size shrink of the kidney. The mouse serum creatinine level had a sharp rise 1 day post-surgery (dps) and gradually recovered by 20 dps, but was still approximately 2-fold higher than the baseline (Fig. S6).

We transplanted 3 × 10^6^ single cell-derived GFP+ uKPC into the incision of the injured kidney. Transplanted kidneys were analyzed 14 days after transplantation. Large-scale engraftment of fluorescent cells into the mouse kidney was observed (Fig. 2G). Frozen sectioning and immunostaining demonstrated incorporation of GFP+ cuboidal epithelial cells into the incised part which form luminal structures in the mouse kidney (Fig. 2H). All GFP+ cells express human-specific nuclei antigen, which proved that the fluorescent tissues were originated from human tissue but not any technical artifact (e.g., autofluorescence). Approximately 3.7% (±1.1%) of the engrafted human cells expressed proliferative marker KI67 (Fig. S7A). All of the GFP+ cells acquired expression of pan-tubular epithelial cell marker PAX8 (Fig. S7B), and most of them expressed proximal tubular cell marker genes SLC22A6 and ATP1A1 (Fig. 2I). Meanwhile, a small number of GFP+ cells (<1%) form lumen structures and express UMOD, which is a marker gene mainly expressed in ascending limb of loop of Helen (LOH) and occasionally the early distal tubules (Fig. 2I). In these GFP+ cells, no expressions of distal tubule cell markers were detected (Fig. S7C). We also performed uKPC transplantation using cells derived from CKD patients and observed similar results (Figs. 2J and S8A).

Of note, the morphology of the lumen structure formed by transplanted human cells was still different from native mouse tubules, suggesting that the mouse kidney microenvironment may not support complete maturation of the human uKPC, probably due to insufficient cross-reactivity of multiple growth factors or cytokines between rodent and human. However, dextran uptake assay revealed the accumulation of fluorescent low-molecule-weight dextran in GFP+ human tubules (Fig. 2K), which suggested that at least some of the regenerated proximal tubular cells could be functionally connected to the glomerular filtrate.

In order to investigate the organ-specificity of uKPC mediated kidney regeneration, we tested whether the stem/progenitor cells derived from other organs rather than the kidney had similar kidney regenerative capacity. We have successfully cultured human lung-derived SOX9+ basal cells (distal airway stem cells) in previous studies (Ma et al., 2018), and here we transplanted an equivalent number of such cells into the injured mouse kidney and found only minimal incorporation. Therefore, the successful engraft of uKPC into the kidney seems to be an organ-specific process (Fig. S8B). Altogether the data above demonstrated that transplanted SOX9+ uKPC (but not SOX9+ cells from other organs) can proliferate and differentiate* in vivo* to gain some properties of tubular cells.

In current study, we presented the first single-cell atlas of adult human urine and identified multiple previously unrecognized cell types. However, as limited by the total analyzed cell number, we could still miss some very low-abundance cell populations. Of note, our current scRNA-seq data is solely based on healthy adults. For those patients with diseases in the renal system, urinary tract system or circulation system, the cell composition of their urine can be very different from healthy people. To overcome these limitations, future work involving a larger cohort will be focused on the comparative analysis of diseased and healthy populations, aiming to develop novel urinary cell-based diagnostic strategies.

One particular cell of interest in urine is the kidney stem/progenitor cell. Previous studies suggested the existence of kidney stem/progenitor cells in adult humans (Maeshima et al., 2003; Sagrinati et al., 2006; Kitamura et al., 2015). Given the large size and complexity of kidney architecture, it is very likely that different types of stem/progenitor cells co-exist in different locations of the kidney and exert distinct functions *in vivo*. Here based on our scRNA-seq analysis data, we identified a *SOX9*+ cell population in adult human urine which we speculated to have progenitor potential. To further investigate the uKPC characteristics, we developed an efficient feeder cell-based “F-Clone” approach to expand such cells. Different from the adult tissue-derived organoid 3D culture system, this feeder cell-based 2D culture system supports the rapid acquisition of a large number of homologous, primitive cells for the purpose of full transcriptomic characterization and subsequent transplantation, providing a realistic option for cell replacement therapies. The ability to isolate KPC from human urine, to genetically modify them, and then to successfully transplant them makes a potentially useful platform for future autologous cell transplantation therapy. Altogether, our research on the special population of endogenous kidney cells improved our understanding of human kidney repair mechanisms and highlights their potential application in personalized regenerative medicine.

## Supplementary Information

Below is the link to the electronic supplementary material.

Supplementary material 1 (PDF 600 kb)
