# Supplementary material for: Single-cell RNA-Seq analysis identified kidney progenitor cells from human urine
Source: Protein Cell. 2021 Jan 9;12(4):305–12. doi: 10.1007/s13238-020-00816-5 (PMC8018990; doi:10.1007/s13238-020-00816-5)
Supplement: Supplementary file 1 — Supplementary material 1 (PDF 600 kb) [file 13238_2020_816_MOESM1_ESM.pdf]

Supplemental materials

Methods

Urine sample collection and processing

For scRNA-seq analysis, urine samples were collected from healthy adult volunteers. Midstream of voided urine specimen was collected for each donor in the morning (not the “first morning”). Samples were centrifuged immediately at the speed of 390 ×g and the cell pellets were washed twice with cold wash buffer including F12 medium, 5% FBS (Hyclone, Australia), 1%P/S (Life, 15070-063), 1% L-glutamine (Life,25030-081) and resuspended in cold phosphate-buffered saline (PBS). Then the cells were passed through a 40-μm Nylon mesh (Falcon, USA) to remove aggregates and casts in the urine. Cell viability was assessed by the exclusion of trypan blue dye. To remove dead cells and cell debris, urinary cells were subjected to fluorescent dyes staining by Calcein AM (Solarbio, C8950-1mg) and DAPI for 30 min at 37°C. Then the cells were washed twice with cold PBS and resuspended in PBS with 2% FBS (Hyclone, Australia) for flow cytometry sorting of Calcein AM stained living cells. Sorted cells were subjected to scRNA-seq immediately.

For the culture of uKPC, urine samples were collected from either healthy adults or patients with chronic kidney diseases, and processed similarly. Patients were diagnosed by K/DOQI clinical practice guidelines. All the human tissues were obtained following SOP with the patient’s consent and approved by the Shanghai Ruijin Hospital Ethics Committee.

Single-cell RNA sequencing and bioinformatic analysis

Single cells were captured and barcoded in 10x Chromium Controller (10x Genomics). Subsequently, RNA from the barcoded cells was reverse-transcribed and sequencing libraries were prepared using Chromium Single Cell 3'v3 Reagent Kit (10x Genomics) according to the manufacturer's instructions. Sequencing libraries were loaded on an Illumina NovaSeq with 2×150 paired-end kits at Novogene, China. Raw sequencing reads were processed using the Cell Ranger v.3.1.0 pipeline from 10X Genomics. In brief, reads were demultiplexed, aligned to the human GRCh38 genome and UMI counts were quantified per gene per cell to generate a gene-barcode matrix. Data were aggregated and normalized to the same sequencing depth, resulting in a combined gene-barcode matrix of all samples. Seurat v.3.1.1 was used for quality control, dimensionality reduction and cell clustering. We removed the low-quality cells with less than 200 or more than 5000 detected genes, or if their mitochondrial gene content was > 25%. Genes were filtered out that were detected in less than 3 cells. This filtering step resulted in 15,760 genes X 1010 cells. We used a global-scaling normalization method ' LogNormalize' to normalize the data by a scale factor (10,000). Next, a subset of highly variable genes was calculated for downstream analysis and a linear transformation (ScaleData) was applied as a pre-processing step. We performed principal component analysis (PCA) dimensionality reduction with the highly variable genes as input in Seurat function RunPCA. We then selected the top 20 significant PCs for two-dimensional t-distributed stochastic neighbor embedding (tSNE), implemented by the Seurat software with the default parameters. We used

FindCluster in Seurat to identify cell clusters. To identify the marker genes, differential expression analysis was performed by the function FindAllMarkers in Seurat with the likelihood-ratio test. Differentially expressed genes that were expressed at least in 25% cells within the cluster and with a fold change more than 0.25 (log scale) were considered to be marker genes. Featureplots were performed by the function Featureplot in Seurat with the default parameters. Threshold of legends was adjusted with the ggpolt2 R package.

Gene Ontology analysis

Gene Ontology (GO) enrichment analysis of differentially expressed genes was implemented by the ClusterProfiler R package. GO terms with a corrected P value less than 0.05 were considered significantly enriched. Dot plots were used to visualize enriched terms by the enrichplot R package.

Single-cell trajectory analysis

Single-cell pseudotime trajectory was constructed by Monocle2 (version 2.12.0) R package. Cells from clusters ' Tubular Cells' and ' Podocytes' were selected for analysis. Genes for ordering cells were identified using differential GeneTest function if they were expressed in ≥ 1% cells, their mean expression value was ≥ 0.1 and qval < 0.01. Based on the ' DDRTree ' method, the data was reduced to two dimensional, and then the cells were ordered along the trajectory.

Cell cycle analysis

Cell cycle analysis was implemented by the Seurat R package. Based on this scoring system, we classified each cell in either G2-M, S or G1/G0 phase using the CellCycleScoring function in Seurat. The cells at different cell cycle classifications were visualized in the tSNE map.

Tubular cells classification analysis

Classification of tubular cells was performed by AddModuleScore function in Seurat. We used a previously published kidney single-cell atlas (Park et al., 2018). Cells were classified by the maximal average expression in these six gene sets , including proximal tubule, loop of Henle, distal tubule, collecting duct principal cell, collecting duct intercalated cell, collecting duct transitional cell. When the scores of six gene sets were both less than 0.15, we considered these cells to be ‘Other’ population. The tubular cells classifications were visualized in the tSNE map.

Isolation, expansion and genetic labeling of uKPC from urine

For uKPC isolation from human urine, the urine samples were centrifuged at 390×g for 10 minutes, the supernatant was carefully removed, and all the cell pellets from one subject were resuspended in ice-cold urine wash buffer (F12 medium containing 5% FBS, 1% Pen/Strep, 20μg/mL Gentamicin and 2.5μg/mL Amphotericin B) and pooled into one 50ml conical tube, followed by extensive washing with urine wash buffer for 5 times. Then each cell pellet was seeded onto the lethally irradiated 3T3

cells feeder layer with modified SCM-6F8 medium in one well of a 12-well plate. Most urine-derived cells did not attach to the feeder layer and were removed by the first medium change on day 3.

Single-cell colonies were selected for pedigree cloning using cloning cylinders and high vacuum grease. For GFP labeling of cultured uKPCs, 293T cells were transfected with pLenti-CMV-eGFP plasmid together with a lentiviral packaging mix (Life Technologies, USA) to produce GFP lentivirus. Lentivirus supernatant was collected, filtered and stored at -80°C until use. Medium containing lentivirus was added to the cell culture together with 10 μg/mL polybrene and incubated for 24 hours, followed by FACS sorting of GFP+ uKPC for subsequent culture.

Histology and immunofluorescence

For cell immunofluorescence staining, cells were fixed by 4% paraformaldehyde, and then incubated with 0.25% Triton X-100 for 8 minutes to permeabilize. For tissue histology and immunofluorescence staining, tissue samples were fixed in 3.7% formaldehyde overnight at 4°C. For cryosection, the fixed tissue was infiltrated with 30% sucrose before embedding, then embedded into the Tissue-Tek O.C.T compound (Sakura, Japan), 5–8 μm sections were prepared using a cryotome (Leica microsystem, Germany). For paraffin section, the fixed tissue was dehydrated by ethanol gradient and processed in an automatic tissue processor, and then embedded into the paraffin blocks. All the samples were sectioned at 5–10 μm thickness using a microtome (Leica microsystem, Germany). Haematoxylin and eosin

(H&E) staining was performed following standard protocol. Immunofluorescence staining was conducted to reveal the protein expression in a standard protocol using the antibodies on paraffin sections, cryosections or glass smears.

Antibodies used for immunofluorescence include: SOX9 (1:200, CY5400, Abways), Ki67 (1:100, 550609, BD bioscience), ATP1A1 (1:50, sc-514614, Santa cruz), AQP1 (1:50, sc-25287, Santa cruz), SLC22A6 (1:200, ab183086, Abcam), Umod (1:200, ab207170, Abcam), AQP2 (1:200, ab199975, Abcam), Calbindin (1:200, 14479-1-AP, Proteintech), SLC12A3 (1:200, ab95302, Abcam), PAX8 (1:200, ab189249, Abcam), SYNPO (1:200, 21064-1-AP, Proteintech), GFP (1:200, ab290, Abcam), GFP (1:500, ab6673, Abcam), HuNu (1:200, ab191181, Abcam), and human specific Lamin A+C (1:200, ab108595, Abcam). Alexa Fluor-conjugated Donkey 488/594 (1:200, Life Technologies, USA) were used as secondary antibodies. In control experiments, the primary antibodies were replaced by IgG.

Cell karyotyping

To arrest human uKPC in mitosis metaphase, cells at 75% confluence were treated with 1 μg/mL colchicines for 7 h and digested into single cells by 0.25% trypsin. Then the cells were incubated with 0.4% KCl at 37°C for 40 min and fixed by 10 mL fixation solution including methanol and glacial acetic acid (3:1) at room temperature for 30 min. The prepared cell suspension was dropped and spread on slides. Samples on slides were treated with 0.0005% trypsin for 5 min and stained with 15% Giemsa (Sigma-Aldrich, USA). Banding patterns on chromosome spreads were checked for

more than 15 mitotic phases.

Detection and calculation of somatic Copy Number Variation (CNV).

We performed an analysis of the CNV from whole genome sequencing (WGS) data. Blood samples were collected from urine sample donors with signed consents and compensation. WGS was performed on genomic DNA isolated from peripheral blood mononuclear cells and the corresponding human uKPCs. All of the libraries were constructed with PCR amplification. We sequenced all sets of libraries on BGISEQ-500 at BGI TechSolutions Co. (BGI-Tech). Control-FREEC was used to CNV and B allele frequency (BAF) profiles and detect regions of copy number gain/loss with default parameters. With peripheral blood mononuclear cell samples from the same individuals as control, Control-FREEC discerns somatic variants from germline ones.

Bulk RNA-sequencing and bioinformatic analysis

Human kidney tissue and uKPCs were analyzed by bulk RNA-sequencing. Duplicate experiments were taken from 2 biological samples. RNA was extracted using RNeasy® Mini Kit (REF# 74104) and a total amount of 3 μg RNA per sample was used as input material for the RNA sample preparations. Sequencing libraries were generated using NEBNext® UltraTM RNA Library Prep Kit for Illumina® (NEB, USA) following manufacturer's commendations and index codes were added to attribute sequences to each sample. Raw data (raw reads) of FASTQ format were firstly

processed through in-house PERL scripts. In this step, clean data (clean reads) were obtained by removing reads containing adapter, reads containing ploy-N and low quality reads from raw data. All the downstream analyses were based on the clean data with high quality. FASTQ files were aligned against the human reference (hg19/hGRC38) genome. Index of the reference genome was built using STAR (v2.5.1b) and paired-end clean reads were aligned to the reference genome. HTSeq v0.6.0 was used to count the reads numbers mapped to each gene, and then FPKM of each gene was calculated based on the length of the gene and read counts mapped to this gene. Differential expression analysis of two conditions/groups (two biological replicates per condition) was performed using the DESeq2 R package (1.10.1). The resulting P-values were adjusted using the Benjamini and Hochberg's approach for controlling the false discovery rate. Genes with an adjusted P-value <0.05 found by DESeq2 were assigned as differentially expressed. Corrected P-value of 0.05 and an absolute fold change of 2 was set as the threshold for significant differential expression. Heatmap were generated using R scripts. PPI analysis of differentially expressed genes was visualized with Cytoscape (v3.2.0) and based on the STRING database.

Mouse kidney injury models

All animal studies were performed under the guidance of Tongji University Association for Laboratory Animal Care and Use. All mouse experiments were performed on both male and female mice and represent a minimum of n=5 mice in all

groups. All mice were randomly allocated to the experimental groups.

For the wedge resection surgery model, the left lateral peritoneum was cut open to expose the left kidney and the kidney artery was clamped with small hemostatic forceps. Then a straight incision was performed on the back of the kidney using a surgical blade to make the resection. After cleaning off the blood, the kidney was sealed with FuAiLe Medical glue (FAL). Then the kidney was located back into the abdominal cavity before the abdomen closing.

Intra-renal transplantation of uKPCs

For intra-renal transplantation, 8-10 week-old NOD/SCID mice (The Jackson Laboratory, USA) were anaesthetized, and a wedge resection was performed. After cleaning off the blood and sealing cut with FuAiLe Medical glue (FAL), GFP labeled uKPCs were injected into the kidney. For each mouse, 3×10^6 cells were used. Mice were sacrificed 1~2 weeks after the transplantation and kidney and blood samples were harvested for further analysis.

Dextran tracing

10,000 MW fixable Alexa Fluor-594 Dextran (Life Technologies, USA) was used to visualize vasculature 14 days after uKPC intra-renal transplantation. 200 μL 5 mg/ml Dextran was injected into mice via the tail vein. 4 hours after injection, the chimeric mice were sacrificed. The kidney samples were collected and fixed with 3.7% formaldehyde (Sigma, USA) at room temperature for 30 minutes. Samples were cryosectioned at 5 μm and observed under a fluorescence microscope (Olympus IX73,

Olympus, Japan) or a confocal microscope (A1R, Nikon, Japan).

Sub-capsule transplantation of uKPCs

8-10 week-old NOD/SCID mice (The Jackson Laboratory, USA) were anesthetized, and the left lateral peritoneum was cut to expose the left kidney. GFP labeled uKPCs were injected into the renal capsule. For each mouse, 3×10^6 cells were used. Mice were sacrificed 1 to 2 weeks after the transplantation and kidney samples were harvested for further analysis.

Isolation and culture of human-lung derived SOX9+ airway basal cells

The bronchoscopic procedure for lung sampling was performed by board certified respiratory physicians using a flexible fiber-optic bronchoscope (Olympus, Japan). Before the bronchoscopy, oropharyngeal and laryngeal anesthesia was obtained by administration of 2 mL of nebulized 4% lidocaine, followed by 1 mL of 2% topical lidocaine sprayed into the patient's oral and nasal cavities. After the bronchoscope was advanced through the vocal cords, 2 mL of 2% lidocaine solution was instilled into the trachea and both main bronchi through the working channel of the bronchoscope. Then a disposable 2-mm brush was advanced through the working channel of the bronchoscope and used to collect airway epithelial cells by gently gliding the brush back and forth 2 times in 4-6 order bronchi in the right or left lobe. To isolate the human DASCs, the brush with samples were cut with scissors into 1 cm pieces. After removing sputum, the brush pieces were directly digested with the

dissociation buffer described above. Specimens were incubated at 37°C for an hour with gentle rocking. Dissociated cells were passed through 70-μm Nylon mesh and then washed twice with cold F12 medium. Cells were plated onto feeder cells in DASC culture medium for lung including DMEM/F12, 10% FBS (Hyclone, Australia), Pen/Strep, amphotericin and growth factor cocktail as previously described (Zuo et al., 2015). Under 7.5% CO2 culture condition, the DASC colonies emerged 3–5 days after plating, and were digested by 0.25% trypsin-EDTA (Gibco, USA) for 3–5 min for passaging. Typically, DASCs are passaged every 5 to 7 days and split at a 1:7 ratio.

Statistical analysis

Block randomization was used to randomize mice and samples into groups of similar sample size. No data was intentionally excluded. Postoperative deaths of animals were excluded from the study. Statistical power analysis was used to ensure an adequate sample size for detecting significant differences between samples. All experiments were assessed by at least two blinded participating investigators. Results are presented as means ± SD. GraphPad Prism (version 7.0a) or R programming was used for data management, statistical analysis and graph generation. Matched results were assessed using Wilcoxon matched-pairs signed-rank test or matched two-way ANOVA. Comparisons among multiple groups were analyzed using Tukey’s multiple comparison test. The exact number (n) of the sample size for each experiment was stated in the corresponding figure legend. Differences with $P \leq 0.05$ were considered statistically significant.

Supplementary References

Park, J., Shrestha, R., Qiu, C., Kondo, A., Huang, S., Werth, M., Li, M., Barasch, J., and Susztak, K. (2018). Single-cell transcriptomics of the mouse kidney reveals potential cellular targets of kidney disease. Science *360*, 758-763.

Zuo, W., Zhang, T., Wu, D.Z., Guan, S.P., Liew, A.A., Yamamoto, Y., Wang, X., Lim, S.J., Vincent, M., and Lessard, M. (2015). p63(+)Krt5(+) distal airway stem cells are essential for lung regeneration. Nature *517*, 616-620.

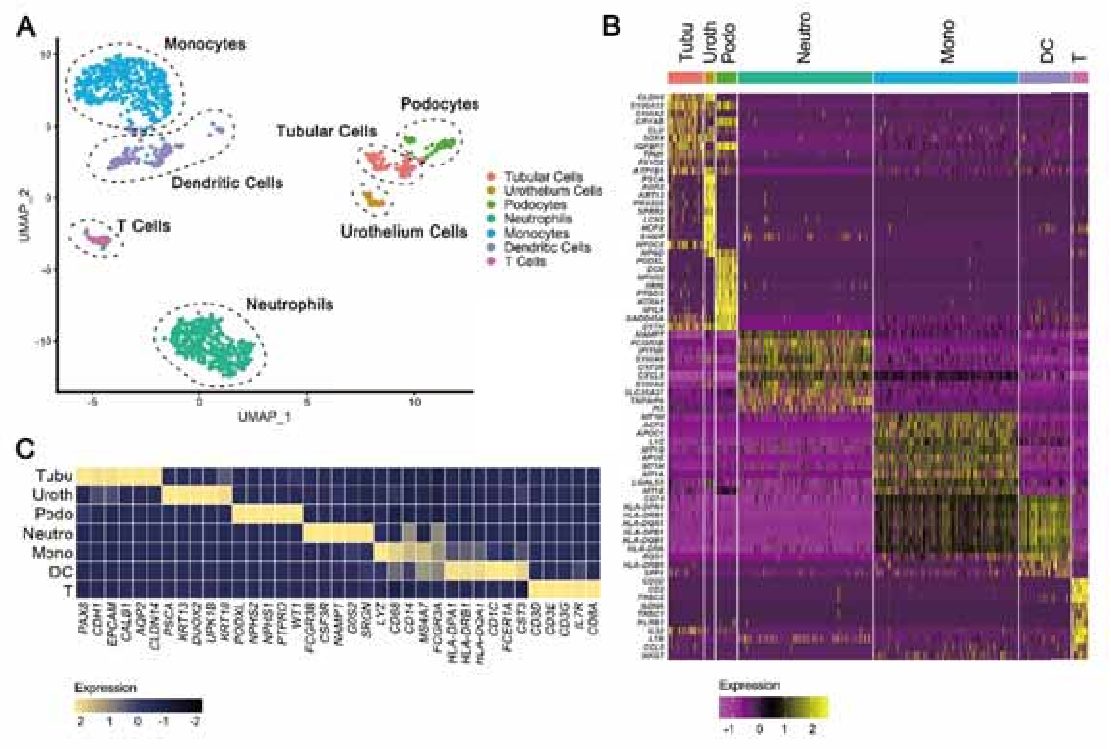

Supplementary Figure 1. Expression of marker gene sets in each cell cluster

(A) Uniform manifold approximation and projection (UMAP) plot of the unsupervised classification of urine-derived cells. (B) Heat map of the top ranked genes highly expressed in each cluster. Color scheme is based on z-score distribution from –1 (purple) to 2 (yellow). (C) Heat map of the expression of other representative marker genes across the seven clusters. Color scheme is based on z-score distribution from –2 (blue) to 2 (yellow). Tubu, Tubular Cells; Uroth, Urothelium Cells; Podo, Podocytes; Neutro, Neutrophils; Mono, Monocytes; DC, Dendritic Cells; T, T Cells.

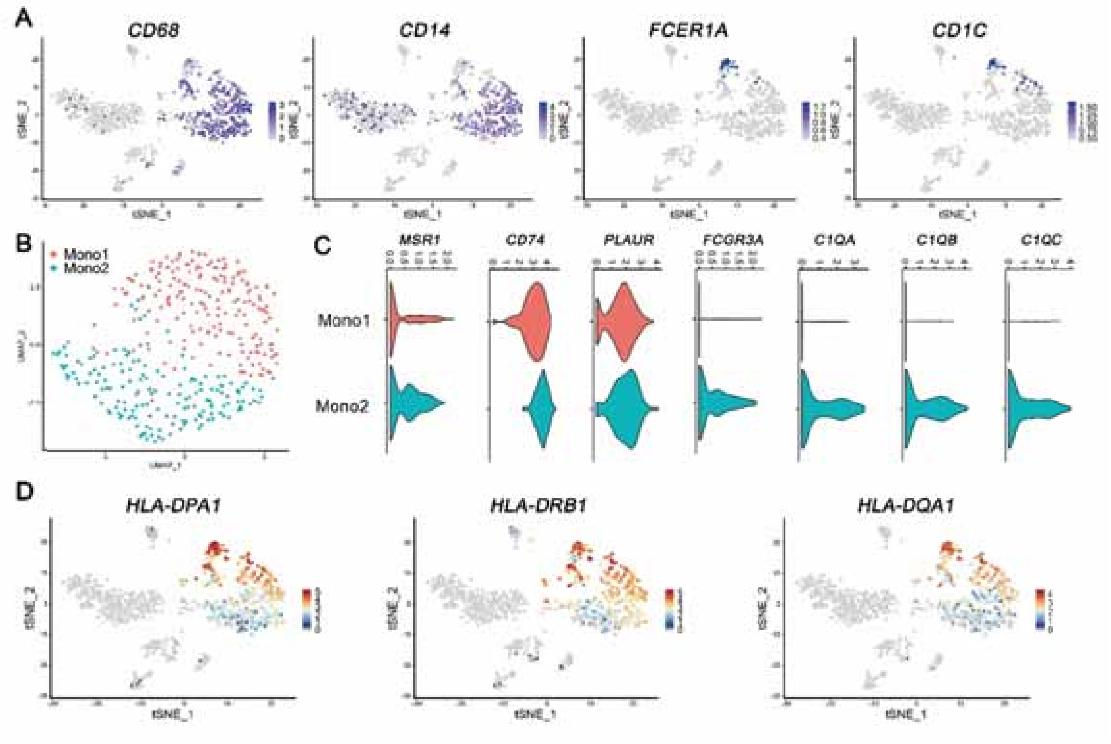

Supplementary Figure 2. Annotation of leukocyte subsets.

(A) tSNE plot highlighting expression of *CD68*, *CD14*, *FCER1A*, *CD1C* in urinary cells. (B) Uniform manifold approximation and projection (UMAP) plot showing two sub-clusters of monocytes after dimensionality reduction. The red dots represent a classic *CD16-* group and the green dots represent a non-classical *CD16+* group. (C) Violin plots showing that *FCGR3A* (*CD16a*) distinguishes monocyte 2 from monocyte 1. *MSR1*, *CD74*, *PLAUR* are expressed in both clusters. (D) tSNE plots highlighting the expression levels of MHC-II receptors *HLA-DRB1*, *HLA-DQA1* and *HLA-DPA1* in urinary cells.

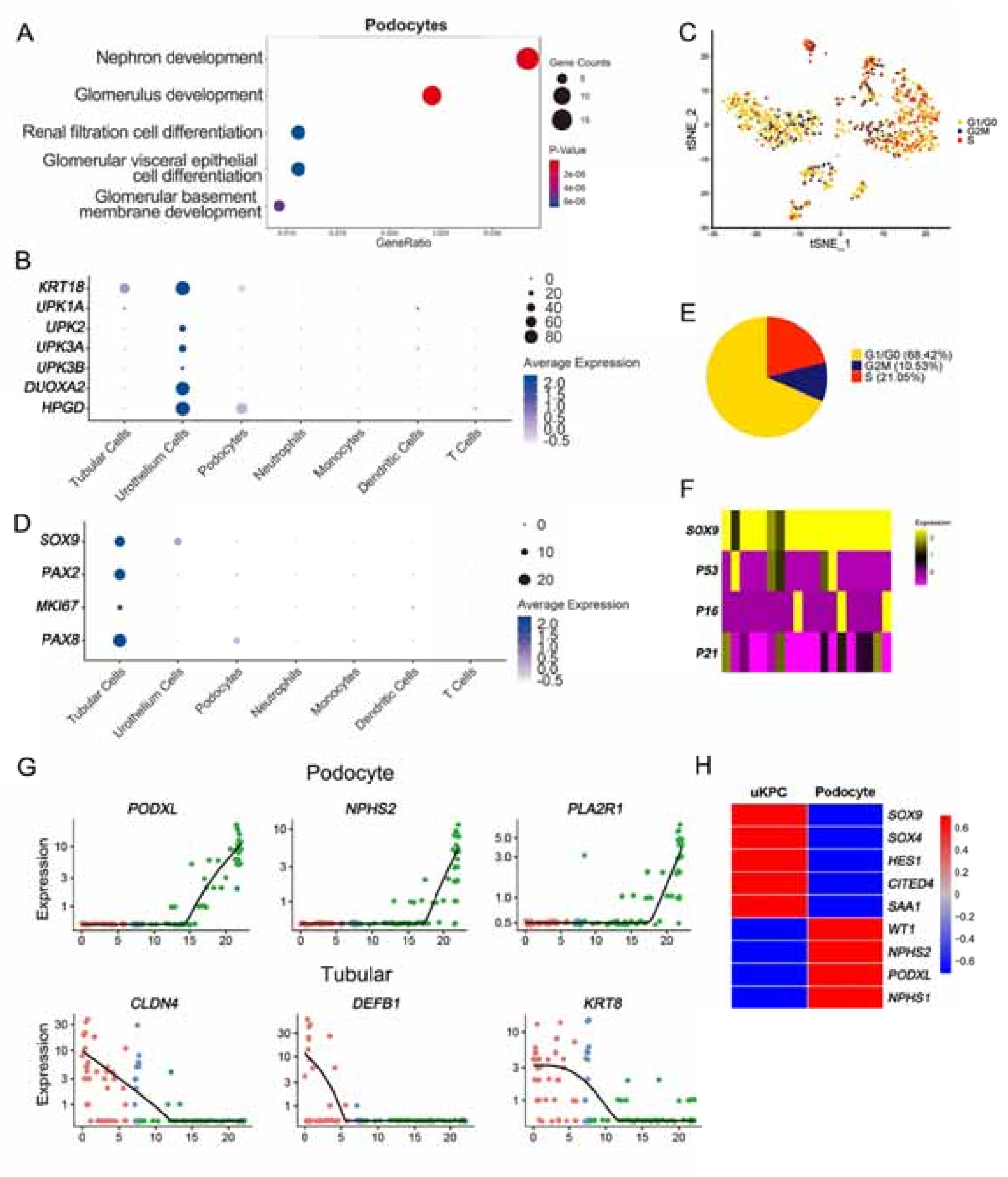

Supplementary Figure 3. Annotation of epithelial cell subsets.

(A) Gene Ontology enrichment analysis of the differentially expressed genes identified in the “Podocytes” cluster. (B) Dot Plot of the expression of specific umbrella cell genes in urine-derived cells. (C) tSNE plot showing the cell cycle status of each cell. (D) Dot Plot of the expression of *SOX9*, *PAX2*, *MKI67*, *PAX8* in urine-derived cells. (E) The proportion of the SOX9+ cells in cell cycle. (F) *P53*, *P16*, and *P21* expression pattern in the SOX9+ cells. (G) Expression dynamics of podocyte marker genes and

tubular marker genes over pseudotime, representing two directions of cell fate determination. (H) Heatmap of differentially expressed gene sets of SOX9- kidney cells and the SOX9+ uKPCs.

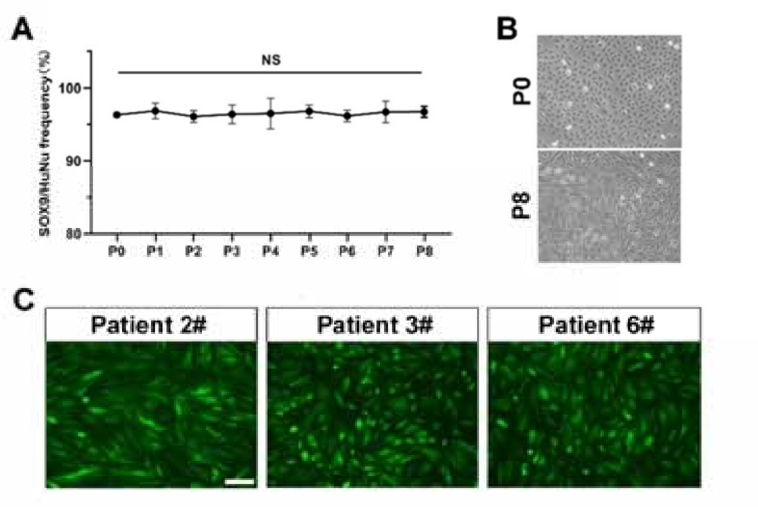

Supplementary Figure 4. Progenitor identity of human uKPCs.

(A) Cultured human uKPC colonies from Passage 0 to Passage 8 were stained with SOX9 and human-specific marker human nuclear antigen HuNu. The frequency of SOX9 expressing uKPCs in all cultured human cells were quantified. n=3 individual assays using independent biological samples. (B) Representative image of the cultured uKPCs at Passage 0 and Passage 8 (n=3). (C) Representative image of the GFP-labled uKPCs derived from CKD patients (n=3). Scale bar, 20 μm.

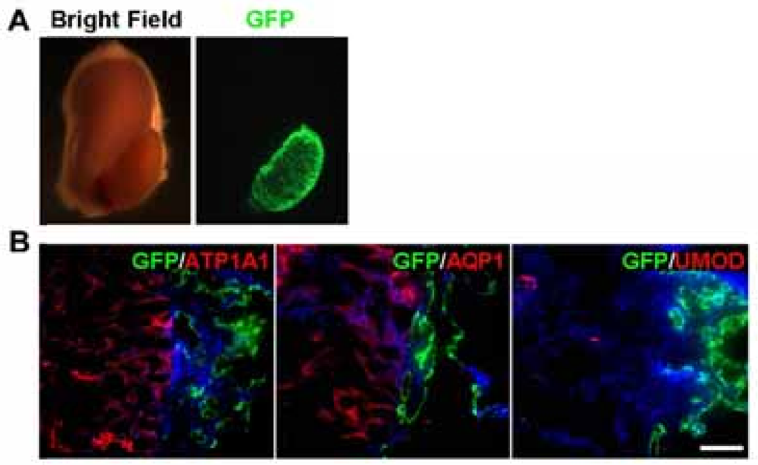

Supplementary Figure 5. Renal capsule transplantation of human uKPCs

(A) Bright-field and direct fluorescence of injured NOD-SCID mouse kidney 14 days after GFP+ uKPC subcapsular transplantation. n=3 independent biological samples.

(B) Frozen sections of transplanted kidney after transplantation followed by immunostaining with GFP and indicated markers. uKPCs at passage 10 were used for transplantation in the representative image. Scale bars, 100 μm.

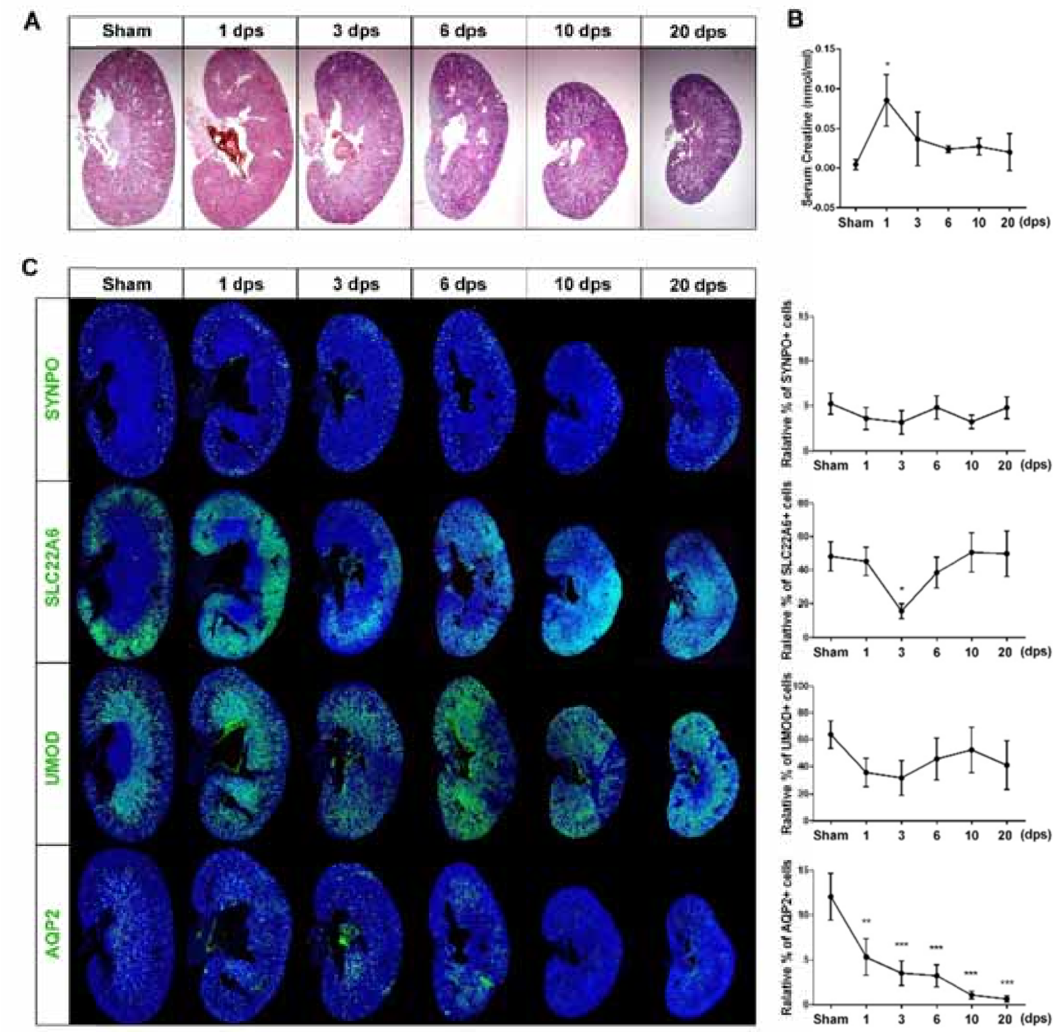

Supplementary Figure 6. Wedge resection model that induces acute kidney injury.

(A) Representative images of H&E stained whole kidney sections at indicated time points. dps, days post surgery. n=3 individual injury experiments using independent biological samples. (B) Serum creatinine level measurement in sham and injured mice. dps, days post surgery. n=3 individual injury experiments using independent biological samples. * P<0.05 compared to Sham control. (C) Representative images and quantification of the immunostained whole kidneys harvested at indicated time points. The expression pattern

and quantification of tubular and glomerulus markers indicated partially restoration of structures. dps, days post surgery. n=3 individual injury experiments using independent biological samples. * $P<0.05$, ** $P<0.01$, *** $P<0.001$ compared to Sham control.

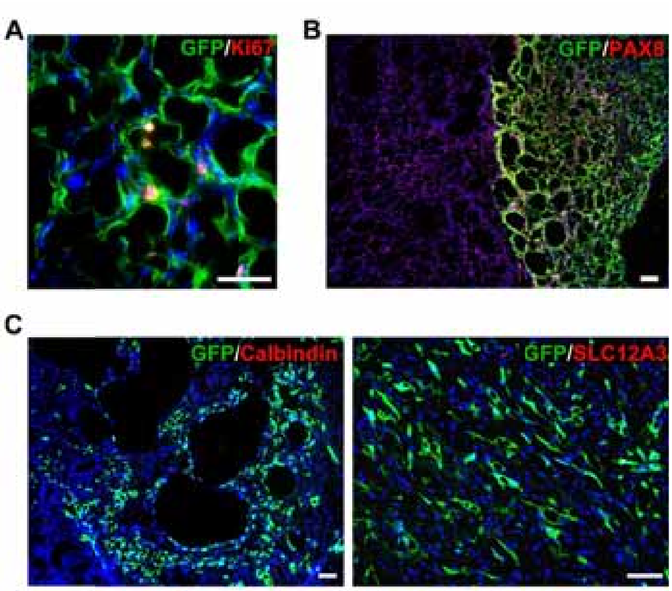

Supplementary Figure 7. Intra-renal transplantation of uKPC

Immunostaining on sections of single cell-derived uKPC transplanted area 14 days after transplantation with PAX8 (tubular epithelium, A), Ki67 (proliferative cell, B), and Calbindin and SLC12A3 (distal tubule, C) antibodies. uKPCs at passage 4 (Ki67 and PAX8) and passage 8 (Calbindin and SLC12A3) were used for transplantation in the representative image. Scale bars, 50 μm.

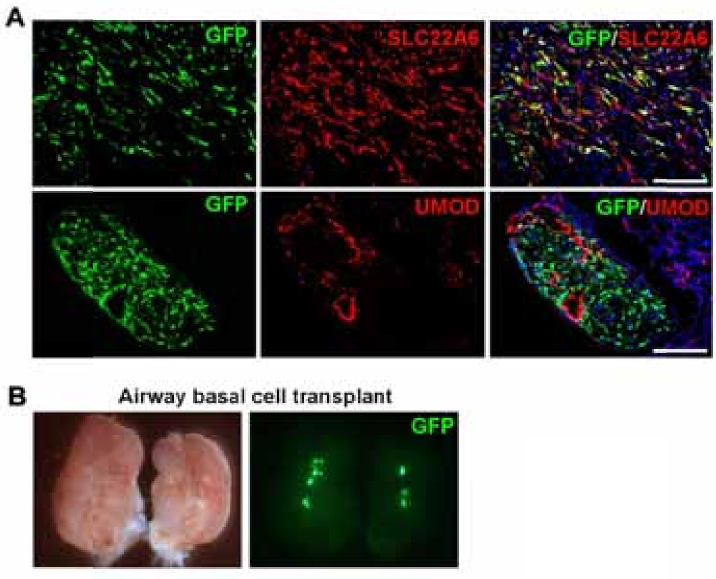

Supplementary Figure 8. Intra-renal transplantation of uKPC derived from CKD patients

(A) Frozen sections of transplanted kidney 14 days after patient-derived uKPC transplantation followed by immunostaining with GFP and indicated markers. uKPCs at passage 7 were used for transplantation in the representative image. Scale bars, 100 μm. (B) Bright-field and direct fluorescence imaging of mouse kidney intrarenally transplanted with GFP-labeled SOX9+ airway basal cell showing minimal engraftment. n=3 independent transplantation experiments. SOX9+ airway basal cells at passage 23 were used for transplantation in the representative image
